# An Irreducible Posterior Fracture-Dislocation of the Shoulder: A Case Report

**DOI:** 10.7759/cureus.24535

**Published:** 2022-04-27

**Authors:** Jacob Shermetaro, Josiah Valk, David Sosnoski, Kelley Brossy

**Affiliations:** 1 Orthopedic Surgery, Beaumont Health, Farmington Hills, USA

**Keywords:** avascular necrosis shoulder, avascular necrosis humeral head, fibular strut graft, orif proximal humerus, proximal humerus fracture-dislocation, irreducible shoulder dislocation, shoulder fracture dislocation, posterior dislocation of the shoulder, locked shoulder dislocation

## Abstract

Posterior fracture-dislocations of the shoulder are exceedingly rare orthopedic injuries. The management of these rare and complex injuries can be challenging from initial presentation through definitive management. Timely diagnosis of these injuries is critical to prevent devastating complications, yet the diagnosis is often delayed. Delays in surgery and poor fracture reduction are associated with a high risk of complications such as avascular necrosis. Additionally, these injuries have the potential to be irreducible. This may occur secondary to osteochondral humeral defects, soft tissue interposition, or entrapment. The long head of the biceps tendon incarceration is one potential block to reduction. Definitive surgical treatment options include open reduction and internal fixation (ORIF) and shoulder arthroplasty. While reoperation rates are higher in patients undergoing ORIF, arthroplasty longevity is a concern among young, active patients with high functional demands. Fibular strut allograft is a useful adjunct when reconstructing complex proximal humerus fractures. We present a case of a 28-year-old male who sustained a significantly comminuted four-part left proximal humerus fracture with an irreducible posterior humeral head dislocation requiring urgent ORIF following a motor vehicle accident.

## Introduction

The shoulder is the most frequently dislocated joint in the body; however, it is most often dislocated anteriorly with or without an associated fracture. Posterior fracture-dislocations of the shoulder are exceedingly rare orthopedic injuries that only account for 2-5% of traumatic shoulder dislocations; these injuries have a reported annual prevalence of 0.6/100,000 [1]. They are most commonly caused by high-energy trauma, electrocution, or seizures resulting from forced flexion, adduction, and internal rotation of the shoulder [2]. The management of these rare and complex injuries can be challenging. Timely diagnosis of these injuries is critical for appropriate early treatment, which may help prevent severe complications such as avascular necrosis [3]. Thorough physical and radiologic examinations are paramount to confirm the diagnosis, which may be missed or delayed in up to 79% of cases [1].

## Case presentation

**Figure 1:**
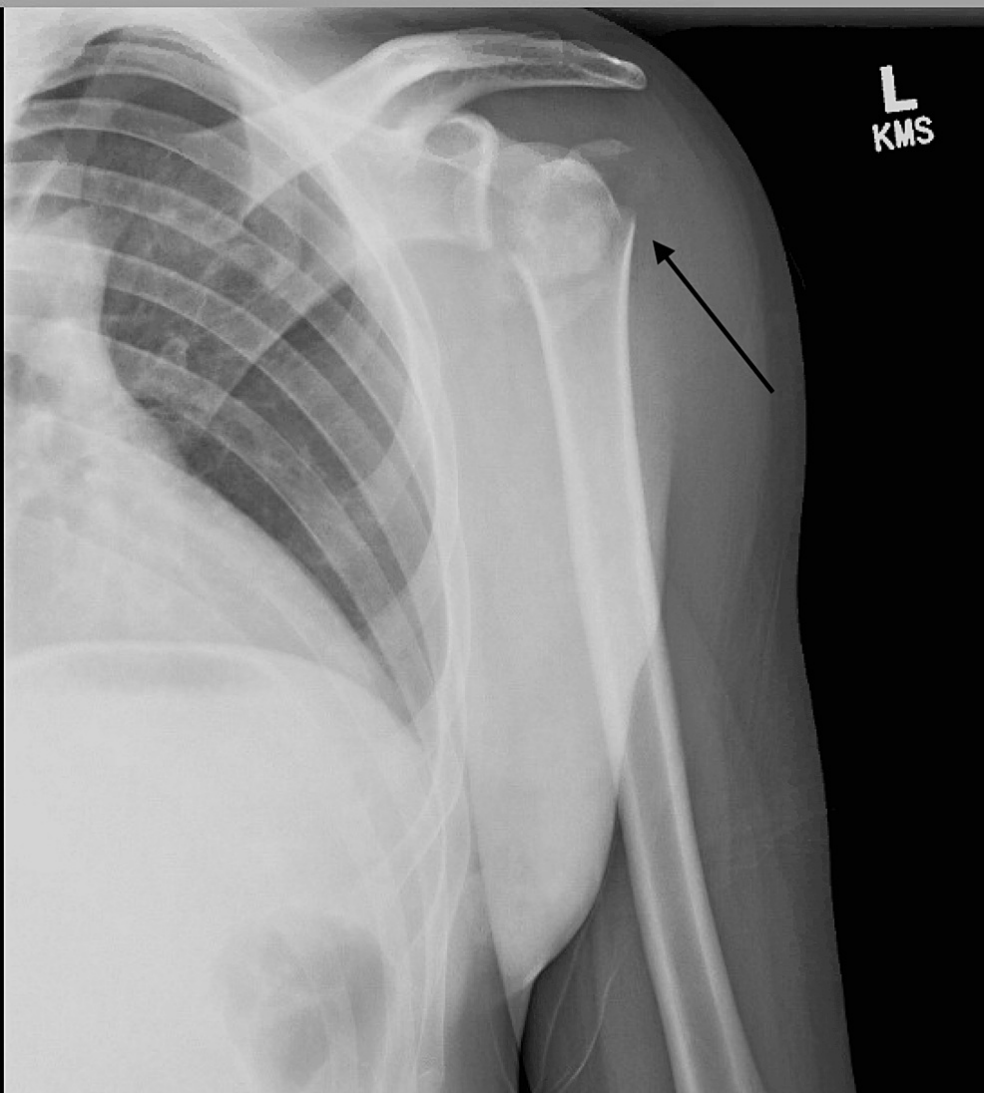
AP radiograph of the left shoulder demonstrating a comminuted proximal humerus fracture with posterior humeral head dislocation AP: anteroposterior X-ray view

**Figure 2:**
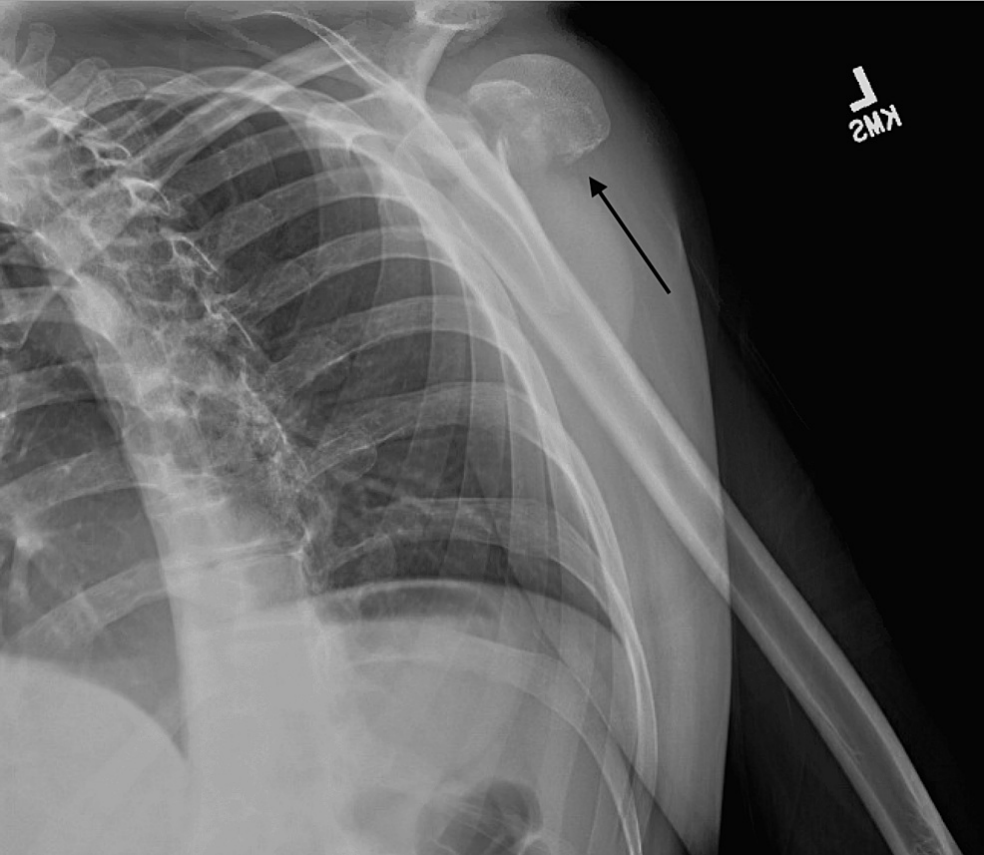
Lateral radiograph of the left shoulder demonstrating a comminuted proximal humerus fracture with posterior humeral head dislocation

A 28-year-old male with a past medical history of seizure disorder presented to the ED with left shoulder pain following a motor vehicle collision. He had been the restrained driver of a vehicle when he had experienced a seizure and subsequently crashed his vehicle into a cement pole. Physical examination of the left upper extremity demonstrated tenderness and swelling on the shoulder. However, his skin was intact and the fascial compartments of the arm and forearm were soft. Motor and sensory testing of his axillary and other major peripheral nerves showed them to be intact. His hand was warm with a palpable radial pulse and brisk capillary refill. Radiographs in the ED demonstrated a significantly comminuted four-part left proximal humerus fracture with posterior humeral head dislocation (Figures 1, 2).

**Figure 3:**
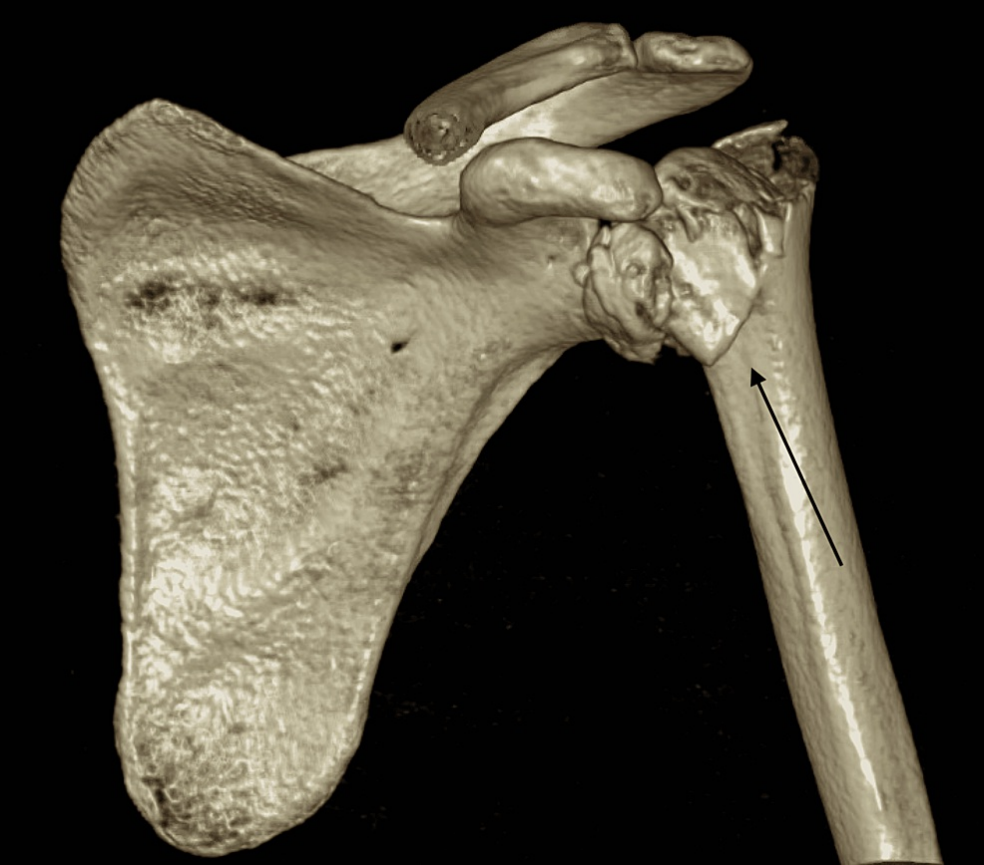
Preoperative CT, anterior 3-dimensional reconstruction view CT: computed tomography

**Figure 4:**
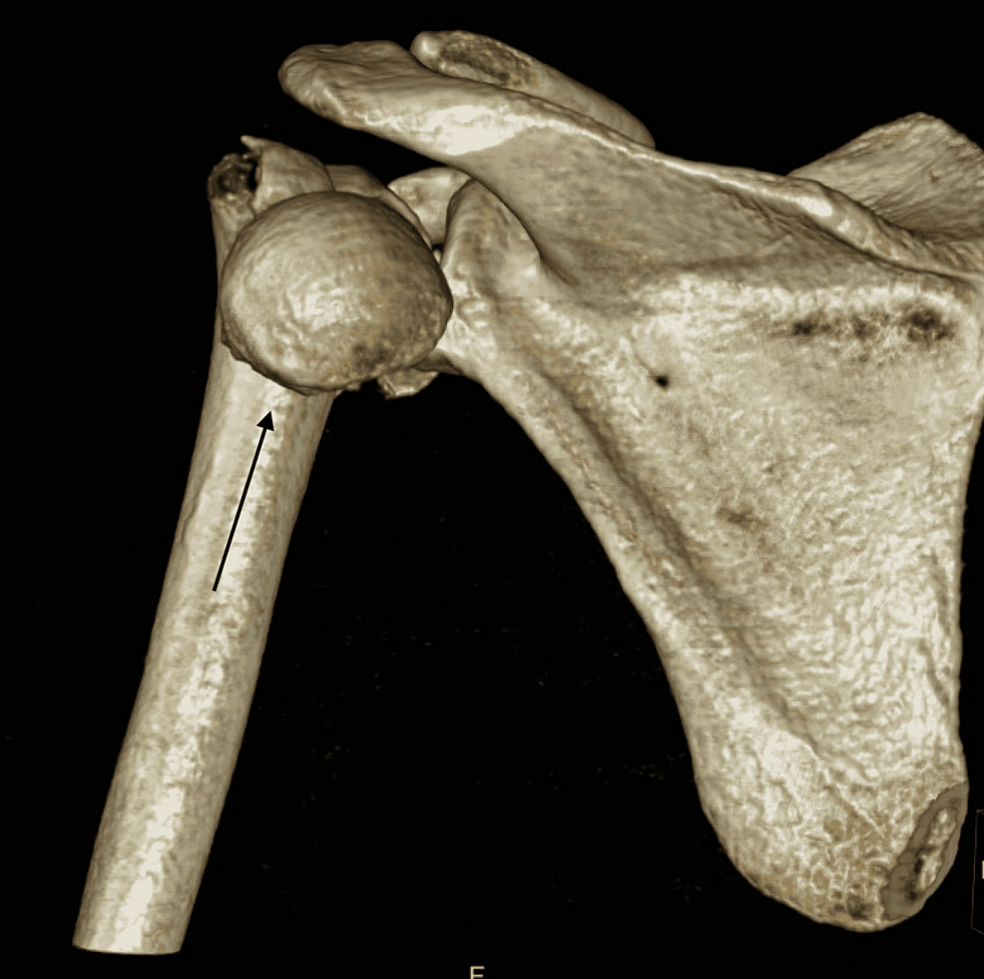
Preoperative CT, posterior 3-dimensional reconstruction view CT: computed tomography

**Figure 5:**
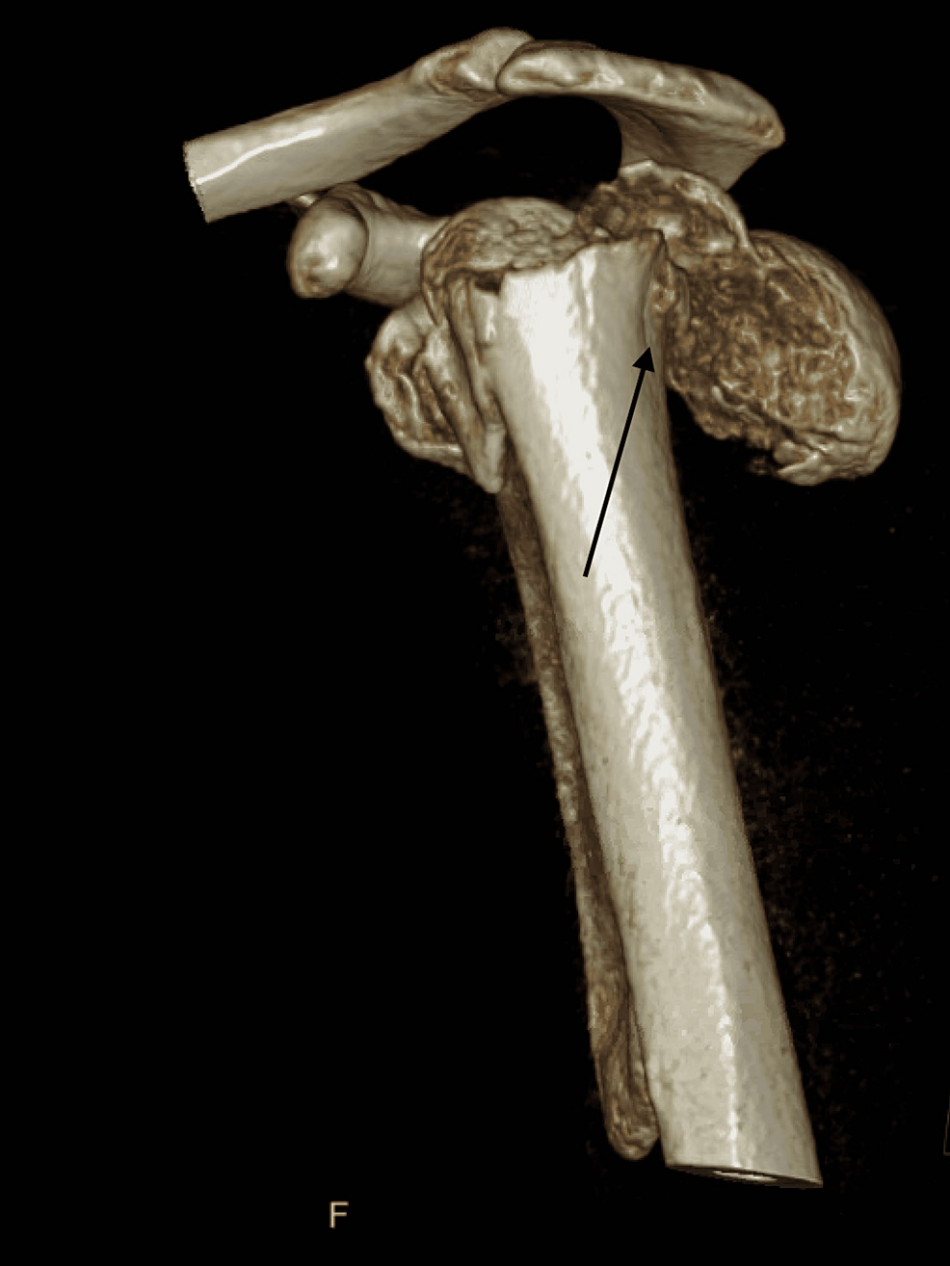
Preoperative CT, lateral 3-dimensional reconstruction view CT: computed tomography

Upon further workup and evaluation, no other major injuries were identified. Even though closed reduction under conscious sedation was attempted in the ED, it was unsuccessful. A CT scan of the left shoulder was obtained for surgical planning (Figures 3, 4, 5).

**Figure 6:**
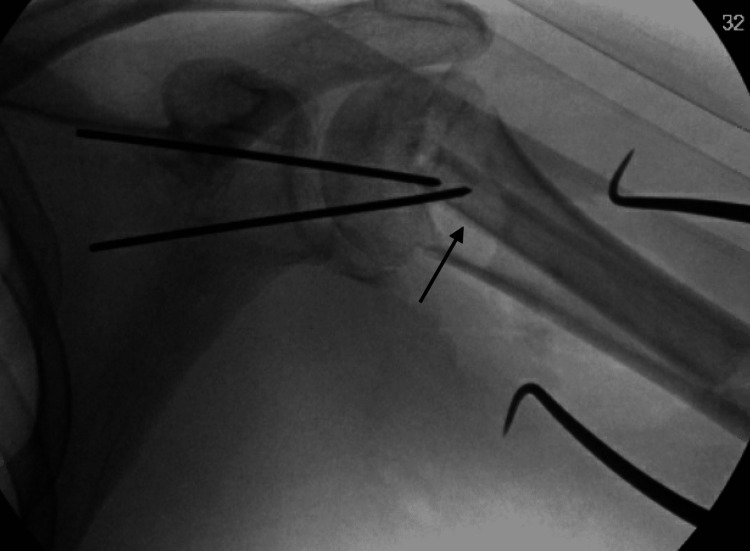
Intraoperative fluoroscopic radiograph demonstrating provisional fixation of the fibular strut graft in the canal of the humeral shaft with pins

The patient was admitted to the hospital and underwent urgent open reduction internal fixation (ORIF) in the operating room on the day of the presentation itself. ORIF was carried out through a standard deltopectoral approach to the shoulder. The long head of the biceps was identified and noted to be traumatized and hyperemic; however, it was not blocking the reduction of the humeral head. This was incised and later tenodesed in a subpectoral fashion. After adequate exposure, the humeral head was noted to be entrapped in the deltoid muscle belly. After extensive debridement and adequate exposure were obtained, the humeral head was ultimately able to be dislodged with a Kocher and reduced. A fibular strut allograft was impacted into the canal of the humeral shaft and the humeral head was perched onto the strut graft. The reduction was held provisionally with Kirschner wires (Figure 6).

**Figure 7:**
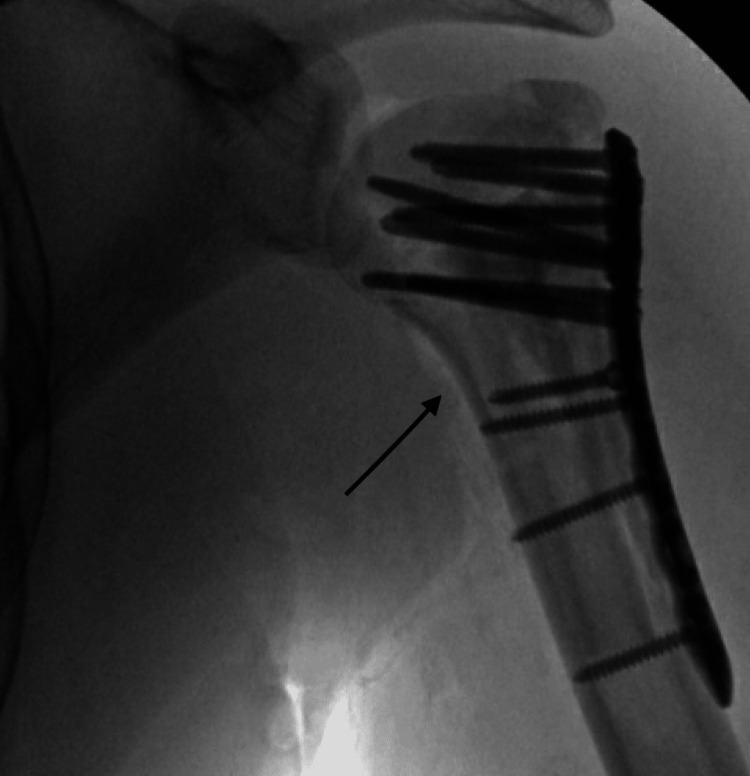
Final intraoperative fluoroscopic radiograph following plate and screw fixation

**Figure 8:**
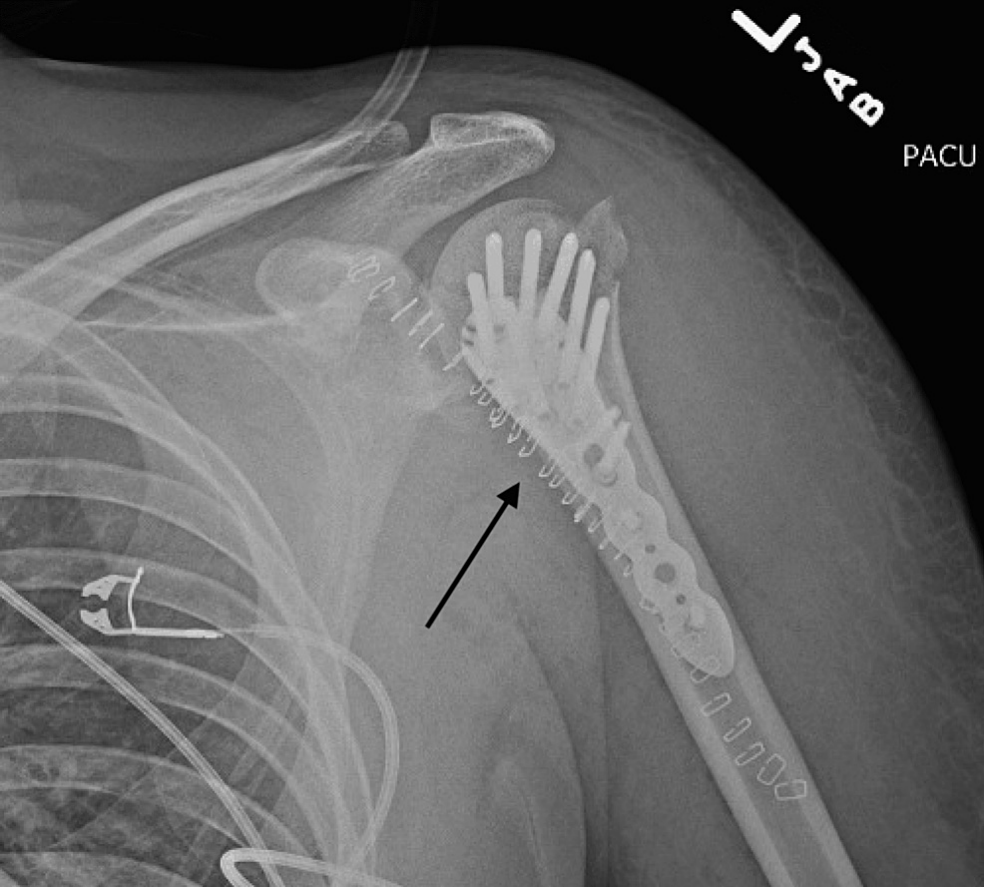
Final postoperative radiograph

Heavy, non-absorbable sutures were placed through the rotator cuff to pull down and help reduce the tuberosity fracture fragments. A proximal humeral locking plate was selected and secured with screws. Final intraoperative fluoroscopic and postoperative radiographs demonstrated acceptable alignment and reduction of the previously described left shoulder fracture-dislocation (Figures 7, 8). No intra-articular screw penetration was noted on intraoperative fluoroscopy.

The procedure was tolerated well by the patient. He was instructed not to bear weight on the left upper extremity, and his shoulder was immobilized with a sling.

The patient's postoperative clinical and radiographic course was closely followed. His surgical neck fracture subsequently achieved union and his greater tuberosity obtained fibrous union. He did develop postsurgical adhesive capsulitis despite an early active range of motion, which was refractory to physiotherapy. At his 10-month postoperative visit, the patient was noted to have an active and passive range of motion of 90 degrees of forward flexion, 90 degrees of abduction, and 5 degrees of external rotation and internal rotation. He elected to undergo removal of the hardware and manipulation under anesthesia. Following this procedure, and one year from the index procedure, the patient had an active range of motion of 100 degrees of forward flexion, 100 degrees of abduction, and 15 degrees of internal and external rotation. His passive range of motion was 150 degrees of forward flexion, 120 degrees of abduction, and 25 degrees of internal and external rotation. At this time, his anteroposterior (AP) and scapular Y radiographs demonstrated the union of the surgical neck with the incorporation of the fibular graft and fibrous union of greater tuberosity. He continues to work with physical therapy and has no imminent plans for surgery.

## Discussion

Posterior shoulder fracture-dislocations are uncommon orthopedic injuries with peak incidences seen in adult male patients between 20 and 49 years of age and elderly patients over the age of 70 years [1]. A timely and accurate diagnosis of this type of injury is essential for its appropriate management. Delayed surgery (more than 48 hours after injury) and poor fracture reduction are associated with a high risk of avascular necrosis [3]. Diagnosis is often delayed in up to 50% of cases upon initial presentation [4]. A thorough history and physical examination are essential aspects of orthopedic trauma management. The majority of delayed or missed diagnoses may be a result of inadequate radiographic assessment [1]. When injuries are suspected on the shoulder and radiographs are warranted, at least one AP view and one lateral view must be obtained. The authors recommend a three-view series of AP, scapular Y, and axillary. Often, an axillary view is exceedingly difficult to obtain in the setting of fracture due to the pain experienced when abducting the arm. In such cases, a Velpeau lateral view should be considered. A CT scan is another option to be considered; however, due to high cost and radiation, it should only be used when a complete shoulder series is deemed inadequate.

These injuries are commonly associated with bony or soft tissue injuries. Bony injuries can include reverse Hill-Sachs lesions or compression-type fractures around the anterior humeral head because the humeral head impacts the scapular glenoid rim during dislocation. Fractures of the proximal humeral tuberosities, anatomic, and surgical neck are also commonly associated. Highly comminuted four-part fracture dislocations present their own set of challenges with regard to treatment. These injuries have the potential to be irreducible. They may occur secondary to osteochondral humeral defects, soft tissue interposition, or entrapment. Interposition and incarceration of the long head of the biceps preventing reduction have been described in the literature [2].

The optimal surgical treatment for these injuries is still a matter of debate. Complex fractures that have undergone ORIF have a higher chance of requiring reoperation when compared to an index arthroplasty procedure [5]. Arthroplasty for fracture is often the last resort for young active patients, since they have a high likelihood of multiple lifetime revisions, even with improved component wear characteristics. Hence, early open anatomic reduction and stable internal fixation are the preferred index treatment options in this patient group, with the goal to preserve the native bone stock. One potential surgical adjunct is the use of an allograft fibular strut placed in the humeral shaft through the fracture site to aid in reduction, support the humeral head, and combat large bone voids.

## Conclusions

Posterior shoulder fracture-dislocations are rare orthopedic injuries that require a timely diagnosis as urgent surgical treatment is often indicated. They may be irreducible by closed means with significant soft tissue interposition. Thorough physical and radiographic examinations are pivotal during initial management. Treatment can be challenging and should be performed on a case-by-case basis with consideration given to fracture and patient factors. Generally, ORIF should be considered the first-line option for reconstructable four-part proximal humerus fractures in young active patients. Fibular strut allograft is an excellent adjunct for ORIF, which may aid in reduction and stable fixation.
